# Similarity measurements of B cell receptor repertoire in baseline mice showed spectrum convergence of IgM

**DOI:** 10.1186/s12865-022-00482-8

**Published:** 2022-03-04

**Authors:** Hongkai Wu, Zhichao Zhou, Shi Xie, Rong Yan, Mingxing Gong, Xingui Tian, Zhanhui Wang

**Affiliations:** 1 State Key Laboratory of Respiratory Disease, National Clinical Research Center for Respiratory Disease, The First Affiliated Hospital of Guangzhou Medical University, Guangzhou Medical University, Guangzhou, China; 2 State Key Laboratory of Organ Failure Research, Guangdong Provincial Key Laboratory of Viral Hepatitis Research, Department of Infectious Diseases and Hepatology Unit, Nanfang Hospital, Southern Medical University, Guangzhou, China

**Keywords:** B cell receptor, Antibody repertoire, Baseline mouse, Isotype, Clonotype

## Abstract

**Background:**

The B cell receptor (BCR) repertoire is highly diverse among individuals. Poor similarity of the spectrum among inbred baseline mice may limit the ability to discriminate true signals from those involving specific experimental factors. The repertoire similarity of the baseline status lacks intensive measurements.

**Results:**

We measured the repertoire similarity of IgH in blood and spleen samples from untreated BALB/c and C57BL/6J mice to investigate the baseline status of the two inbred strains. The antibody pool was stratified by the isotype of IgA, IgG and IgM. Between individuals, the results showed better convergence of CDR3 and clonal lineage profiles in IgM than in IgA and IgG, and better robustness of somatic mutation networks in IgM than in IgA and IgG. It also showed that the CDR3 clonotypes and clonal lineages shared better in the spleen samples than in the blood samples. The animal batch differences were detected in CDR3 evenness, mutated clonotype proportions, and maximal network degrees. A cut-off of 95% identity in the CDR3 nucleotide sequences was suitable for clonal lineage establishment.

**Conclusions:**

Our findings reveal a natural landscape of BCR repertoire similarities between baseline mice and provide a solid reference for designing studies of mouse BCR repertoires.

**Supplementary Information:**

The online version contains supplementary material available at 10.1186/s12865-022-00482-8.

## Background

B cells play key roles in humoral and adaptive immune responses. These cells arise from hematopoietic stem cell precursors, express surface B cell receptors (BCRs), and secrete the same proteins as immunoglobulin (Ig) into serum after they differentiate into plasma cells [1]. The enormous diversity of B cells with different BCRs enables them to recognize and bind numerous different antigens, thus ensuring and maintaining host protection.

BCRs comprise paired Ig heavy (IgH) and light chains, with most of their diversity generated by the somatic recombination of individual variable (V), diversity (D, heavy chain only), and joining (J) gene segments during B cell development [2]. Further diversity is introduced by nucleotide trimming at the V-D and D-J joining sites and by template-dependent (P-addition) and independent (N-addition) nucleotide insertions at joined junctions [3]. Another step during which activated B cells become diversified is mediated by somatic hypermutation, which occurs in the germinal center and comprises the selection of B cells with increased affinity for specific antigens [4, 5].

BCRs have three complementarity determining regions (CDRs). CDR1 and CDR2 are encoded in the V gene segment. V(D)J joining processes define CDR3 [6], which often lies at the center of antigen-binding sites, thus serving as a persistent natural identifier of antibody clonality. Furthermore, the antibody isotype of Ig is determined by the IgH constant region [7] and developed by IgH class switching, which occurs soon after the activation of mature naïve B cells and results in a switch from the expression of IgM and IgD to expression of IgG, IgE, or IgA. This isotype/class switch is important for determining the effector function of antibodies [8].

The diversity of a BCR repertoire can be characterized based on either genomic DNA or mRNA sequences isolated from B cell populations [9, 10] using high-throughput sequencing (HTS). Studies of the BCR repertoire have generally focused on IgH and its CDR3, which is the most variable and critical for determining antigen-binding specificity [11].

Inbred mouse strains such as BALB/c (BAL) and C57BL/6J (C57) are major sources of antibodies used for diagnostic, reagent, and therapeutic applications. They are also widely used in studies of humoral immune responses and B cell-related mechanisms. Experimental mice and HTS of the BCR repertoire have been used to engineer monoclonal antibodies [12–14], analyze the immunization status [15–18], characterize and position B cell subtypes [19, 20], and assess the effects of the spaceflight on antibody repertoires [21–23]. Information on the mouse BCR repertoire is also useful for network analyses of the sequence architecture [24, 25].

However, few studies have focused on the baseline BCR repertoire of experimental inbred mice. A high proportion of V gene sequences is not identical between BAL and C57 mice, indicating that these strains have distinct germline-focused antibodies [26]. A study of redundant IgG repertoires in unimmunized BAL mice amplified using three distinct PCR primer sets did not find CDR3 overlap among 3 of the 50 most abundant sequences [27]. The extent of disparity among untreated mice is an essential pillar of BCR repertoire investigations, as numerous individual differences greatly affect the detection of true signals caused by experimental changes in internal or external environment factors, particularly when such changes introduced by various factors are minor. To clarify this issue, we systematically assessed similarities in the IgA, IgG, and IgM repertoires from the blood and spleen of baseline BAL and C57 mice at 5–7 weeks of age, which are widely used in immunization experiments. We first identified qualified sequences as clones, and then grouped them by 3 levels, V/D/J gene usage, CDR3 amino acid (aa) clonotype and clonal lineage. For each level, the similarity of the BCR repertoire between individuals was investigated. The nature of repertoire convergence was characterized to improve our understanding of antibody diversity in crossed baseline mice, which may aid in designing of experiments involving the mouse BCR repertoire.

## Results

### Sequencing output and V/D/J gene usage

The mouse blood and spleen samples were subjected to two rounds of amplification with immediate sequencing. The first round included three BAL mice, and both the blood and spleen samples were amplified for technical replication and sequenced. The second round included samples from five BAL mice and five C57 mice without technical replication. The time interval between the two rounds of experiments and their respective purchases of mice was ~ 6 months. Both batches of mice were purchased at 4–6 weeks old and were acclimatized for 1 week before starting the experiments.

Additional public datasets of mouse IgH repertoires were collected for comparative analysis. Data from the spleens of untreated BAL (n = 4, 11 weeks old) and C57 (n = 5, 8–10 weeks old) mice were obtained from the ArrayExpress microarray database (E-MTAB-5349; E5349). Blood data from untreated C57 mice (n = 5, 8–10 weeks old) sampled at -10 days (or before infection) from a longitudinal study were also obtained from ArrayExpress (E-MTAB-8585; E8585). Compared with the data sequenced herein, the two public datasets differed in that the IgH variable region was amplified using multiple primers and that only the isotype of IgM was the target of amplification, furthermore, in the experiment for E5349, naïve B cells in the spleens were isolated first and used for library construction.

We classified IgA/G/M isotypes in our sequencing data according to their specific primer sequences (Additional file 1: Fig. S1). The proportion of IgG was lowest, accounting for 1.18 ± 0.51% and 2.98 ± 2.06% in the blood and spleen samples, respectively. The proportions of IgM were much higher than those of IgA in the BAL spleen but relatively similar in the blood samples. Two rounds of experiments revealed a consistent trend.

Additional file 2: Table S1 summarizes information on the qualified sequencing throughput and number of unique clonotypes in each subset of clones identified according to the levels of V/D/J genes and CDR3 aa/nt. The spleen IgM data showed that E5349 yielded averages of ~ 6.3 × 10^6^ pairs of clone reads and ~ 5.3 × 10^5^ unique CDR3 aa types; the corresponding values obtained from our sequencing dataset were ~ 6.8 × 10^5^ and ~ 1.2 × 10^5^, respectively. The blood IgM data showed that E8585 yielded an average of ~ 1.3 × 10^5^ pairs of clone reads and ~ 6.2 × 10^4^ unique CDR3 aa types; the corresponding values in our sequencing dataset were ~ 4.9 × 10^5^ and ~ 4.1 × 10^4^, respectively. Briefly, the numbers of qualified sequencing outputs in our dataset were lower in the spleen and higher in the blood compared to those in the public dataset. The differences in output numbers between individuals in the E5349 and E8585 datasets were relatively large.

**Fig. 1:**
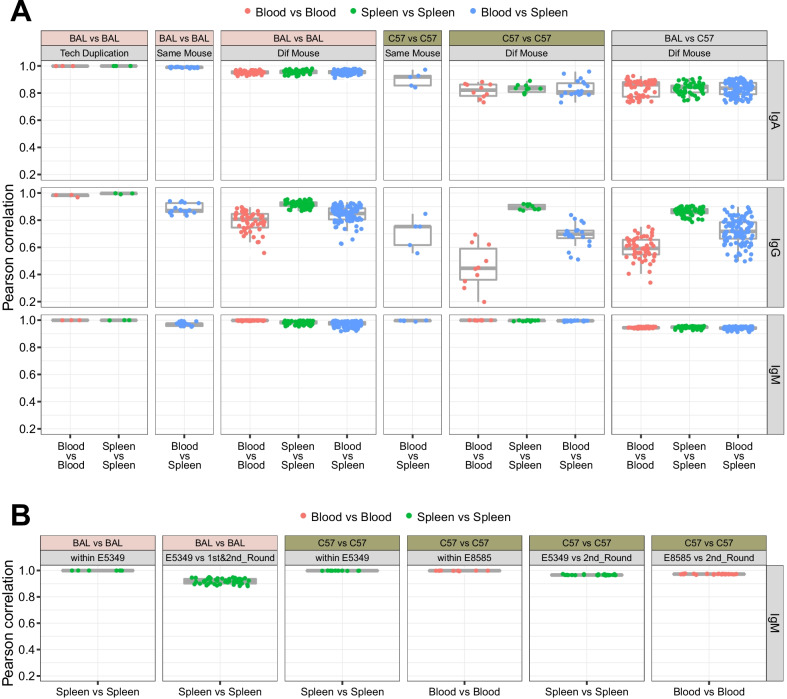
Pearson correlation index of gene frequencies. Indexes were computed for each pair of individuals in first and second rounds (**a**) and for each pair of individuals in E5349 and E8585, with the results of comparing corresponding data from first and second rounds (**b**). Tech Duplication and Dif Mouse indicate comparisons between technical duplications and different mice, respectively

The frequencies of the V/D/J genes were calculated for each subset. Additional file 3: Fig. S2 shows the frequency of each gene in IgM. Gene usage was more uniform for the D and J genes than for the V genes between batches. We used the frequencies of the V/D/J genes in pairwise samples to calculate Pearson correlation indexes to quantify the correlation of gene usage (Fig. 1). Higher index indicated the frequencies of the two samples on the same gene were more consistent. The indexes among technical duplications were almost 1, and the correlations were closer among IgMs than among IgAs and IgGs. The IgA and IgG indexes were higher between BAL individuals than between C57 individuals. The IgG indexes were higher between spleen samples than between blood samples in both BAL and C57 individuals. Values for IgM were also high between individuals within E5349 and E8585. The indexes computed through the public data coupling with our lab sequencing data revealed that gene usage remained closely correlated, with the lowest value of 0.8810 in the comparisons between the spleens of BAL individuals (Fig. 1b). In the gene usage level, the results showed the reproducibility of the experiment, and suggested that the correlation of IgM among individuals was close to the correlation of technical duplication.

### Assessment of sequencing depth and repertoire diversity

**Fig. 2:**
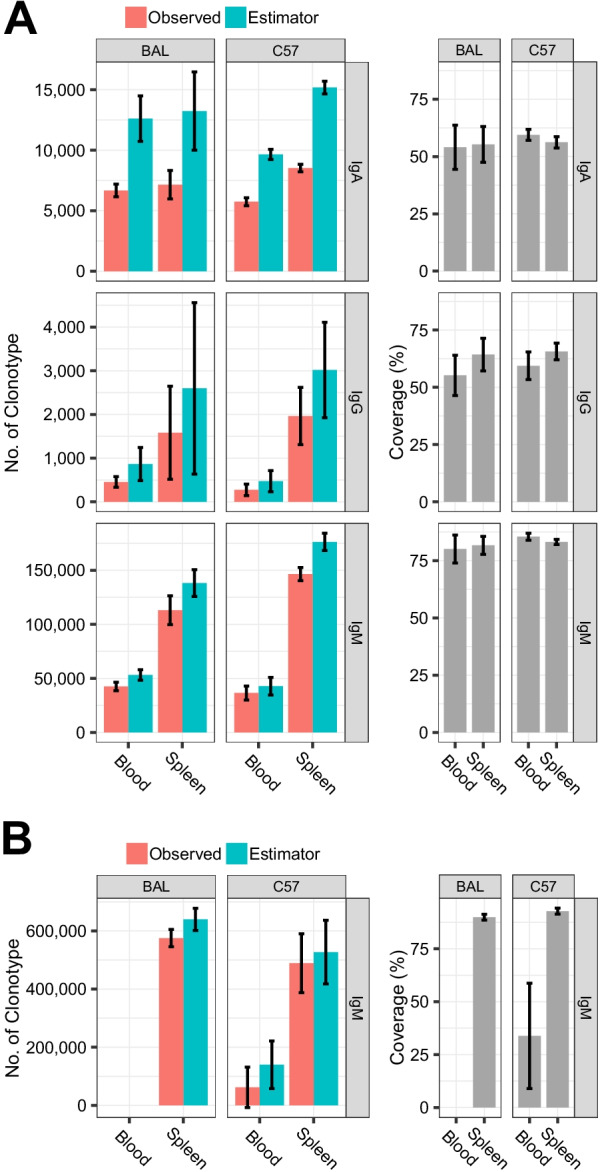
Number of clonotypes and coverage of observed clonotype over estimated full richness. The results were computed from in-house sequencing data (**a**) and E5349 and E8585 public datasets (**b**). Left panel, numbers of observed clonotypes and estimated richness. Right panel, ratios (%) of observed clonotype of estimated full richness. Columns and error bar represent means ± SD. We used one dataset with numerous clones for technical duplication

We identified clones from the sequencing reads and classified them into clonotypes with identical CDR3 aa sequences. An increasing range of clone proportions in a subset was bootstrapped to calculate the number of clonotypes. We then estimated clonotype richness through extrapolation based on Chao’s estimator formula (Additional file 4: Fig. S3). The raising curves indicated that an increased sampling depth would yield few additional clonotypes; thus, sampling depth could be assessed by observing whether the curve reaches the plateau. The actual numbers and estimated richness of clonotypes were summarized for each subset, and coverage was computed to show the ratios (%) of clonotypes detected among the estimators (Fig. 2). The actual or estimated richness values of the CDR3 aa clonotype was higher in the spleen than in the blood. The average sequencing depth covered 56.27%, 61.10%, and 82.60% of the IgA, IgG, and IgM clonotypes, respectively, in our in-house data. The coverage of IgM in public data of the spleen was 91.36%. Separate evaluations of our in-house BAL and C57 data revealed a relatively wide range of IgG richness in the spleens, and individual differences were smaller in the spleen and blood for IgM than for IgA and IgG coverage (Fig. 2a). The results suggest that the spleen had more clonotypes than the blood, and that the sequencing depths of IgM were relatively sufficient which covered similar percentages of estimated richness among different individuals.

**Fig. 3:**
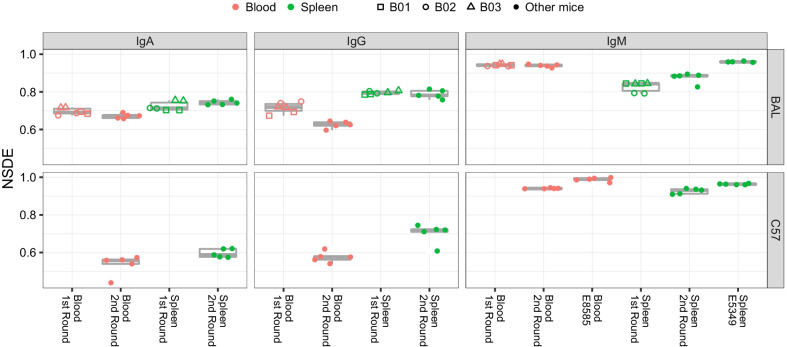
NSDE of CDR3 aa repertoire. B01, B02 and B03 identify mice with technical duplications

We computed the normalized Shannon diversity entropy (NSDE) of the clonotype frequencies. This diversity index ranged from to 0–1, with a higher value indicating that the repertoire had more evenness. Figure 3 shows that the NSDEs of IgM subsets were higher than those of IgA and IgG. The NSDEs of IgA or IgG were higher in the spleen than in the blood, and the difference in medians was most pronounced in the IgG of C57 mice (0.7194 vs. 0.5760, *p* = 0.0158, Wilcoxon rank sum test). The NSDEs of IgM was higher from E5349 or E8585 than from our in-house data. Except for IgM in the blood, the NSDEs differed between the first or second rounds. Among them, the median values of blood-IgG in BAL showed the largest differences (0.7188 vs. 0.6252, *p* = 0.0043, Wilcoxon rank sum test). The results indicated that the CDR3 aa repertoires of IgM were more even than those of IgA and IgG, and the repertoires of IgA and IgG from the spleen were more even than from the blood. The difference of NSDE between the first or second rounds indicated that the evenness of the repertoire was inconsistent when the batch of experimental mice was not identical, particularly with respect to IgG in blood.

### Repertoire similarity in the level of CDR3 amino acid clonotype

We examined the variations among abundance profiles of CDR3 aa clonotype after observing high similarity between individuals at the V/D/J gene level. However, the Pearson correlation index was low at the aa level. If not from technical duplication, the indexes were in the interquartile range of -0.0124 (Q1)—0.0773 (Q3), indicating poor similarity among mice and the limitation of using this scale for comparisons. Thus, we measured similarity between pairs of repertoires using the abundance data of each CDR3 aa clonotype to compute Morisita-Horn similarity index (MHSI) (Additional file 5: Fig. S4). Values ranged from to 0–1 and a value closer to 1 indicated more similar profiles of the CDR3 aa abundance between two samples.

**Fig. 4:**
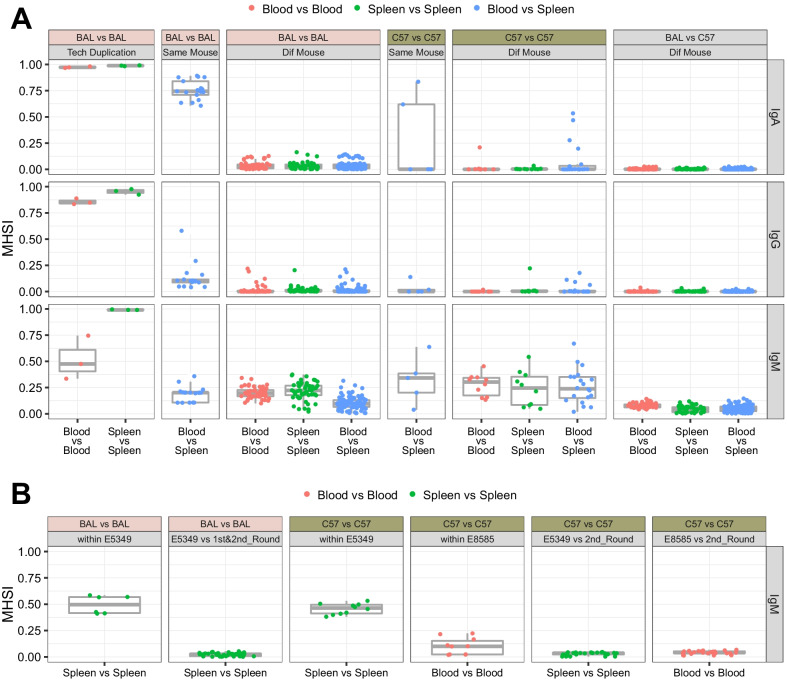
MHSI of CDR3 aa repertoire. Indexes were computed using each pair of subsets in first and second rounds (**a**) and pairs of individuals in E5349 and E8585, with the results of comparing corresponding data from first and second rounds (**b**). Tech Duplication and Dif Mouse indicate comparisons between technical duplications and different mice, respectively

We classified MHSIs into categories (Fig. 4) as previously performed for Pearson correlation index of V/D/J genes. Most MHSI values for the technical duplication in our sequencing data were overwhelmingly high compared with the non-technical duplication results, except for the values in blood vs. blood IgM which were relatively low. MHSI was higher for IgM than for IgA and IgG, regardless of the sample type between different mice within the same strain (Fig. 4a). For example, the median values between the spleens from two BAL mice were 0.2233 for IgM and 0.0303 for IgA (vs. IgM, *p* = 9.103e−10, Wilcoxon signed rank test, the same test method followed) and 0.0061 for IgG (vs. IgM, *p* = 9.638e−10) and those between blood samples from two C57 mice were 0.3030 for IgM, 0.0005 for IgA (vs. IgM, *p* = 0.0020), and 0.0000 for IgG (vs. IgM, *p* = 0.0020). The similarity of IgM in the spleens was even closer among mice in the public datasets (median values: BAL and C57 spleens, 0.4961 and 0.4637; C57 blood, 0.1008). However, when the data of each individual were paired with the corresponding IgM data of each individual from our experiments, the MHSIs were low, ranging from 0.0170 (Q1) to 0.0373 (Q3) in the spleen and 0.0348 (Q1) to 0.0531 (Q3) in the blood (Fig. 4b). The results indicated that the similarity of IgM CDR3 aa profile among individuals of the same animal source was higher than that of IgA and IgG.

We investigated the similarity between the blood and spleen samples from the same BAL mouse and found that the median value was higher for IgA (0.7447) than for IgM (0.2017; vs. IgA, p = 1.526e-05) and IgG (0.1020; vs. IgA, *p* = 1.526e−05). In two of five C57 mice, MHSI values (> 0.6) were determined from the blood vs. spleen IgA (Fig. 4a). The results indicated high convergence of the IgA repertoire between the blood and spleen samples from a single mouse.

**Fig. 5:**
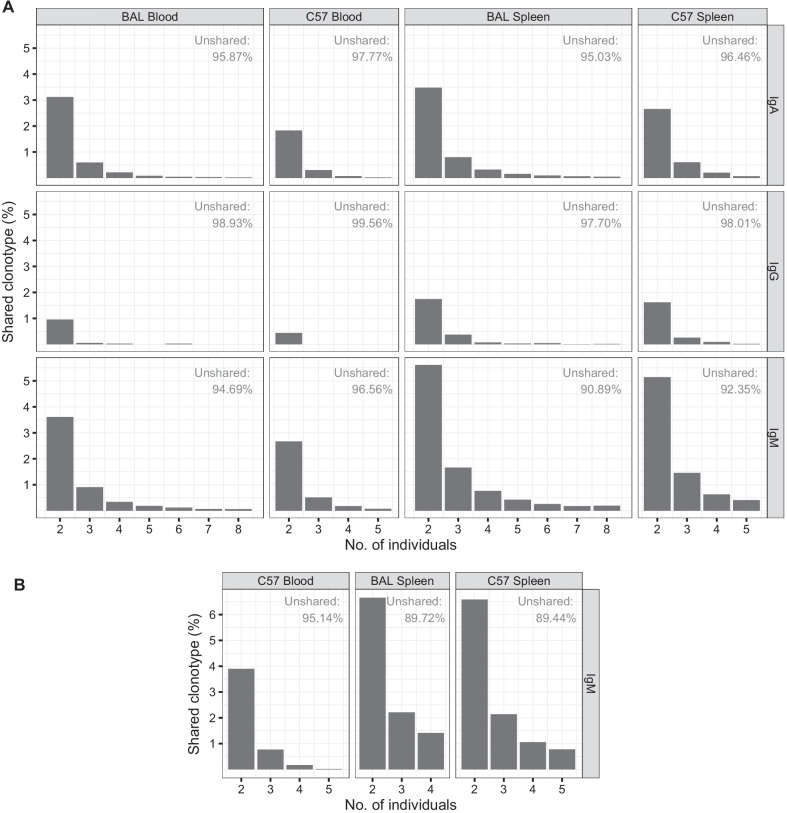
Ratio (%) of shared clonotypes among individuals. In group of same isotype, same sample type and one mouse strain, columns indicate ratios (%) of total clonotypes in group shared among individuals. Number in upper right corner of group indicates ratios (%) of total clonotypes detected in only one mouse. The results were derived from in-house sequencing data (**a**) and E5349 and E8585 dataset (**b**). We used one dataset with numerous CDR3 aa clonotypes for technical duplication

We analyzed the shared clonotypes in the same sample type within the strain to further evaluate the similarity among individuals (Fig. 5). A large proportion of clonotypes was found in only one individual, and very few clonotypes were found across all individuals in a group. The result indicated CDR3 aa clonotypes were shared poorly among individuals. However, the prevalence of shared clonotypes remained higher for IgM than for IgA and IgG. The ratios (%) of unshared clonotypes were consistently higher in the blood than in the spleens of BAL and C57 mice, and those of shared clonotypes across various individuals were higher in the spleen than in the blood, indicating that the CDR3 aa clonotypes were more convergent in the spleen than in the blood.

### Somatic mutation assessment and comparison

For surveying the somatic mutations, we fist explored the criteria for identifying a clonotype that was mutated from another. In each individual isotype dataset, CDR3 nucleotide (nt) clonotypes with the same V and J genes and CDR3 nt length were grouped together. In each group, we calculated the CDR3 nt sequence similarities between each clonotypes and all other clonotypes, and for one clonotype, the other clonotype with the highest sequence similarity was defined as its nearest neighbor, then plotted the 2D distribution of the clonotype number according to the CDR3 nt length and the similarity of their nearest neighbor (Additional file 6: Fig. S5). The abundance of sequences in the experimental data indicated hot spots of sequences that had nearest neighbors with ≥ 95% similarity and of sequences with nearest neighbors that had ~ 55–85% similarity for IgA and IgG, and ~ 70–90% for IgM. The high numbers of clonotypes in the vertical segment of similarity ≥ 95% were also distributed over a wide range of nt lengths (Additional file 6: Fig. S5a–f). A boundary between the two hot spots was not observed in the IgM results from E5349/ E8585, but the abundance was still high in the similarity area of ≥ 95% in the spleen, (Additional file 6: Fig. S5h). Based on this finding, sequences with nearest neighbors that were ≥ 95% similar implied that they belong to the same clonal lineage, whereas ~ 55–90% similarity implied that they got the values by random sampling and do not belong to a clonal lineage. Thus, the same V and J genes and CDR3 nt length along with a cut-off of 95% CDR3 nt sequence identity could serve as the criteria for determining whether two sequences belonged to the same clonal lineage, that is, a somatic mutation directly derived from another.

**Fig. 6:**
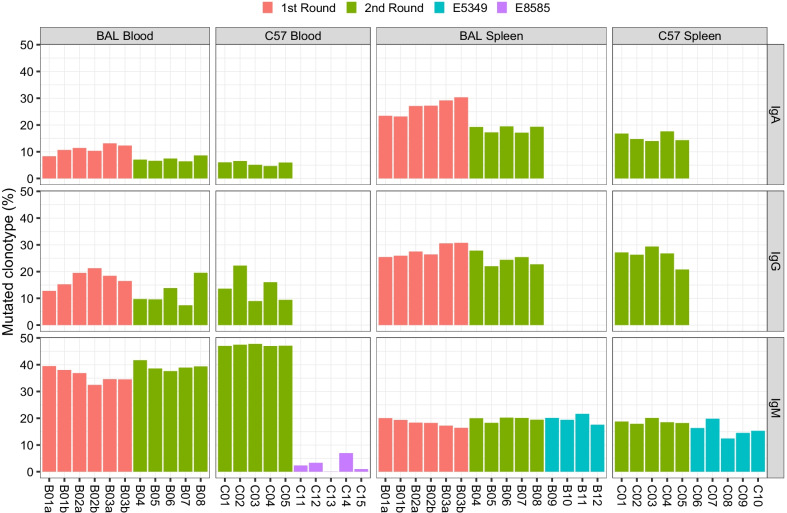
Ratios (%) of clonotypes in somatic mutation lineages. Pairs of technical duplications are marked with letters a and b

After these criteria were established, we created clonal lineages that included at least two clonotypes which could be grouped together under the criteria. In each individual isotype, the total number of clonotypes in all clonal lineages was counted, and the ratio (%) relative to the total number of clonotypes represented the proportion of mutated clonotypes in the snapshot (Fig. 6). We found more mutated IgA and IgG clonotypes in the spleen than in the blood, this trend was reversed in IgM except for in the blood data from E8585. Meanwhile, in the two rounds of experiments, the ratio (%) of IgA was higher in the first round than in the second round. The median IgA levels were 11.02% and 7.02% in the blood (*p* = 0.0087, Wilcoxon rank sum test), and 27.13% vs. 19.25% in the spleen (*p* = 0.0043, Wilcoxon rank sum test).

**Fig. 7:**
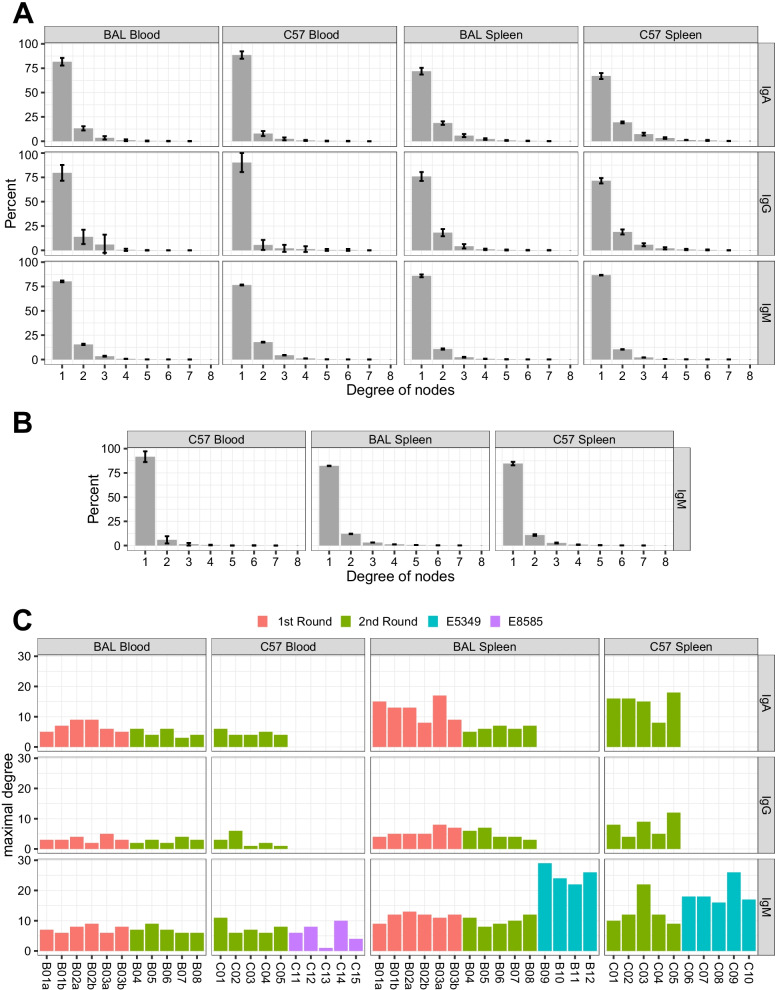
Degree of nodes in somatic mutation network. Distributions of node degrees with clonal lineages computed from our in-house data (**a**) and public E5349/ E8585 dataset (**b**). Columns, ratios of up to 8 degrees; error bars, means ± SD. We used only one set of data with numerous CDR3 nt clonotypes for technical duplication. Colored columns indicate maximal degrees in samples (**c**) of different batches. Pairs of technical duplications in mice are marked with letters a and b

Each clonal lineage was considered as a network; CDR3 nt clonotypes among them were regarded as nodes, and links between a node and its somatic mutation counterparts were considered as edges. The number of edges emitted from a node is the degree. So, a larger degree reflected more somatic mutation counterparts of a clonotypes on the node. We calculated the degree for each clonotype. Thereafter, the aggregated distribution of these numbers showed a full view of the number of mutations in the clonotypes (Fig. 7a, b). The error bars on the columns for IgM were shorter than those for IgA and IgG except for C57-blood in the public data. Our sequencing data showed that the proportion of IgA and IgG clonotypes with ≥ 2 edges was larger in the spleen than in blood, whereas that of IgM was larger in blood than in the spleen (Fig. 7a).

While an exceedingly small proportion of clonotypes belonged to the section with a degree > 8, the maximal degree found among clonotypes in each sample was plotted (Fig. 7c). The maximal degree for IgM was larger than that for IgA and IgG, and in the spleen, it was large than in the blood. The difference in the maximal degree was detected between experimental batches in the BAL spleens. In this subset, the IgA median of the first round was 13, compared with 6 in the second round (*p* = 0.0077, Wilcoxon rank sum test). The median IgM of our data was 11, compared with 25 in those of E5349 (*p* = 0.0045, Wilcoxon rank sum test).

In summary, the results of somatic mutation analysis indicated that IgM was with greater uniformity of the degree distribution than IgA and IgG among individuals, the network of IgA and IgG clonal lineages was denser and more expanded in the repertoire in the spleen than in the blood, and batch differences occurred in the proportion of mutated clonotypes and the maximal degree.

### Repertoire similarity at the clonal lineage level

Next, we established cross-individual lineages for further investigating the repertoire similarity at the clonal lineage level. In each isotype of each mouse strain, we combined all clonotypes of blood and spleen samples of all individuals using our in-house sequencing data. The criteria for clustering mentioned above were applied to establish 6 groups of cross-individual lineages for 3 isotype of BAL and C57 respectively. The numbers of nodes in the lineages were counted, and the distribution showed that ~ 70% of lineages had only 2 nodes (Additional file 7: Fig. S6). The relatively large lineages with no less than 3 nodes were retained, and nodes occurring in any sample of the blood or spleen were traced back by the identical V, J gene and CDR3 nt sequence according to their isotype and strain (Additional file 8: Table S2).

**Fig. 8:**
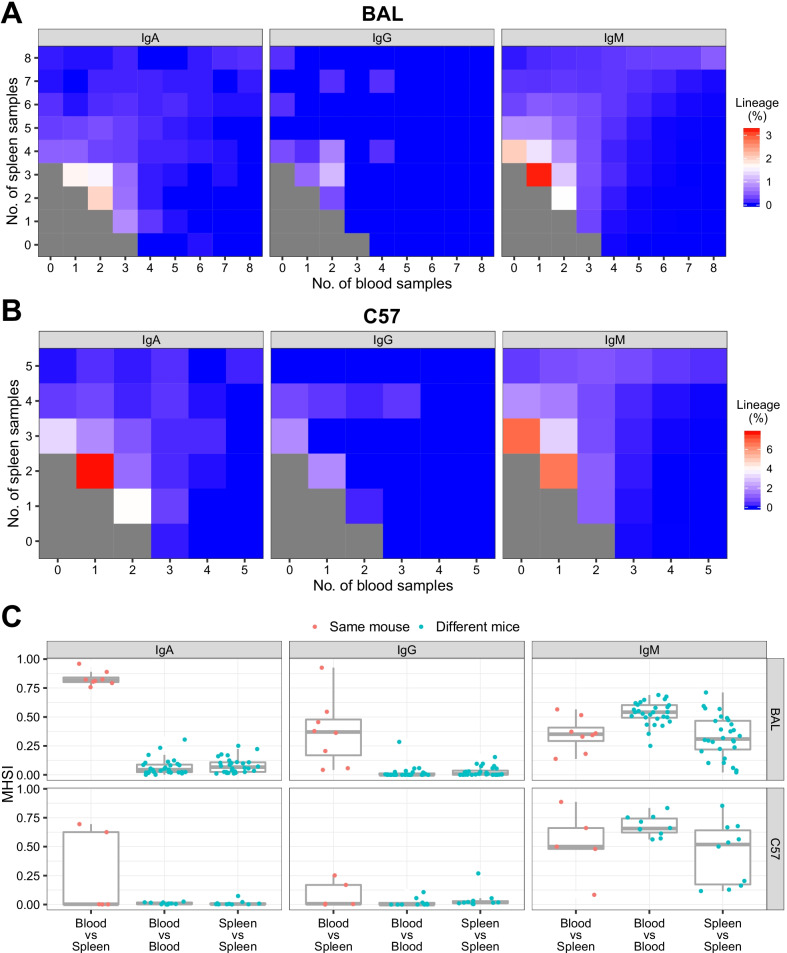
Repertoire similarity measured by cross-individual clonal lineages with no less than 3 nodes. The percentage distributions of lineages appeared in different number of blood and spleen samples were calculated from in-house sequencing data of BAL (**a**) and C57 (**b**). For the clarity of the color display, the percentages of small sample numbers are omitted (grey squares in **a** and **b**). Different colors of the dots indicate the MHSI values were computed from the same mouse or different mice (**c**). One dataset with numerous CDR3 nt clonotypes was used for technical duplication

In order to investigate the sharing of the cross-individual lineages, when at least one node in the lineage appeared in an individual sample, it was considered that the lineage existed in the sample, thus, a lineage could be assigned to multiple individual samples. For each lineage, we counted the number of individual blood or spleen samples in which the lineage existed. We then computed the percentages of lineages in each group, relative to the different number blood samples and spleen samples. Both in BAL and C57, the distributions of the percentages showed that lineages of IgA and IgM were shared in more individual samples of blood and spleen compared to IgG, and the percentage distribution of IgM was more even than that of IgG (Fig. 8a, b). The result also showed that when the lineages were shared among relatively small number of blood samples (e.g. < 5 blood samples in BAL or < 4 blood samples in C57), they were shared in larger numbers of spleen samples, but when the sample types were interchanged, this phenomenon was not observed (Fig. 8a, b). This indicated that lineages in the groups were shared better in the sample of spleen than blood. We also performed this analysis for more large lineages of those with no less than 5 nodes, the results showed the consistent trends in the lineage distribution (Additional file 9: Fig. S7a and b).

To further evaluate the similarity of the cross-individual lineages among individuals, we considered the abundances of the lineages in the samples additionally. The frequencies of nodes in the lineages in each sample were summed to obtain abundance information for the lineages. We then calculated the MHSIs for each pair of samples as performed for the CDR3 aa clonotypes. The results showed that the MHSIs of IgM between individuals were higher than those of IgA and IgG both in BAL and C57 (Fig. 8c). For example, the median values between the blood samples from two BAL mice were 0.5416 for IgM and 0.0441 for IgA (vs. IgM, *p* = 7.451e−09, Wilcoxon signed rank test, the same test method followed) and 0.0007 for IgG (vs. IgM, *p* = 7.451e−09) and those between spleen samples from two C57 mice were 0.5185 for IgM, 0.0046 for IgA (vs. IgM, *p* = 0.0020), and 0.0178 for IgG (vs. IgM, *p* = 0.0039). As observed for the CDR3 aa clonotypes (Fig. 4), the blood vs. spleen MHSIs of the same BAL mouse in IgA were very high with a median of 0.8170, whereas the medians in IgG and IgM were 0.3704 (vs. IgA, *p* = 0.0078) and 0.3512 (vs. IgA, p = 0.0078), respectively (Fig. 8c). Again, we computed the MHSI for more large lineages with no less than 5 nodes (Additional file 9: Fig. S7c). The results were similar with Fig. 8c indicating the high similarity of the cross-individual lineages of IgM between individuals and of IgA within individuals of BAL.

## Discussion

It is known that BCR repertoires in mice are with high individual variability, while the extent of difference in untreated inbred mice has not been specifically measured. Studies typically deployed control vs treatment designs to determine the specific characteristics of BCR profiles under the influence of designated factors. However, when evaluating the differences between very similar treatments, such as immunizations of close viral strains, the extent of background variability in baseline mice may affect this discrimination. Furthermore, when using a longitudinal design of multi time point sampling in mouse experiments to avoid individual differences, the amount of blood collected is generally not sufficient to support subsequent experiments, and the traumatic operation affects the subject's subsequent immunological status. Our particular investigation of the BCR repertoire in baseline inbred mice provided a comprehensive view of the repertoire similarity between mouse individuals to aid in the assessment of experimental designs.

We identified the similar profiles of many aspects. In summary, comparing with current associated studies, our results show the consistent trends of high similarity in V/D/J gene usage and unique profiles of CDR3 aa clonotype. Due to the focus on the baseline object and some different analysis methods used, as well as the stratification of isotypes, our results provided new and more complete information.

The V/D/J usage in IgM closely correlated. The median Pearson correlation of V gene frequencies in IgM amplified from naïve B cells in the C57 spleen was 0.98 (0.88–0.99), even in pairs of mice coupled from a pool of untreated and infected mice [15]. However, this value was 0.16 ( − 0.38 to 0.67) [15] for IgG amplified from the plasma cells of bone marrow. This was much lower than our blood vs. blood results showing values of 0.45 (0.36–0.59) in C57 and 0.81 (0.75–0.85) in BAL, which were the lowest values in the sample type comparison but computed from the pool of V, D and J genes (Fig. 1a).

Our results agree with those of several studies that have found unique CDR3 aa repertoires in mice. Based on total mRNA sequences from spleen tissue of naïve control C57 mice, only < 1.03% of the CDR3 aa clonotypes of heavy chain in one individual was shared across three mice [22]. The ratio (%) of public clones (fraction of unique clones each mouse shared with the other individuals) in IgM derived from blood was ~ 0.3% in a cohort of five naïve mice before infection [18]. The ratio (%) computed from the number of shared clonotypes between two individuals divided by the minimum number of clonotypes in the two repertories among untreated mice was only 14% in IgM from the spleen and 0% with a relatively large SEM in IgA [15]. Although the similarity of the CDR3 aa profiles was low, our MHSI results indicated that IgM had relatively high values compared with IgA and IgG in both sample types of both strains (Fig. 4). For example, the median MHSI of IgM in blood was 0.3030 (0.1753–0.3425, Q1–Q3) for C57 and 0.1972 (0.1675–0.2262, Q1–Q3) for BAL. In fact, in our previous study of IgM sampled from human blood [28], we found that the highest time-point-pair MHSI (time point setting: before and 7 days after 0, 1, and 6 months of hepatitis B vaccination) of the same participant (n = 9) was only 0.0489 (0.0219–0.0858, Q1–Q3) in memory B cells (data not shown). This meant that the similarity of the IgM CDR3 aa profiles between different experimental inbred mice greatly exceeded the similarity between different time points in one human individual.

In an analysis of the repertoire similarity between tissues, a study revealed that the overlap between the IgG or IgA CDR3 repertoire of memory B cells from the spleen and bone marrow in each of three immunized C57 mice was significantly smaller than that determined by random extraction from clonotypes initially observed in pairs of tissues [20]. This segregation of repertoires of the spleen and bone marrow suggested tissue-specific compartmentalization. In our study, the MHSI of IgA computed from the blood vs. spleen from one mouse was relatively high in all BAL and in two of five C57 mice (Fig. 4a). This indicated that IgA had a relatively good overlap between blood and spleen samples.

Other repertoire characteristics reported here also agreed with the results of previous studies. For example, the Shannon evenness (NSDE-like calculation) was close to 1 in blood IgM of untreated C57 mice [17], which is similar to our finding (Fig. 3). In the network of repertories, the number of CDR3 nodes, edges, and clonotypes in the largest component revealed the reproducibility and robustness of the antibody repertoire architecture [25]. Our calculation of network parameters (Figs. 6, 7) also supported this robustness in the same batch of mice at baseline, particularly for IgM.

There were several methods for the sampling depth assessment or the establishment of clonal lineage. Understanding the limitations and differences of these methods can help interpret our results.

By applying a non-linear regression method, the previous study combined 38 individual’s repertoires to estimate that the number of CDR3 aa clonotypes in C57 mice was 10^13^, which covers 95% of the entire richness, and all observed clonotypes covered 42% of clonal diversity [15]. Chao’s method was applied in our study. We considered only one individual and a single isotype, thus, the estimated total clonotype was much smaller. Among the C57 results, the largest sizes were in the IgM of the spleen at 1.8^5^ and 5.2^5^ in our in-house and E5349, respectively. The observed clonotypes covered 81% and 91% of the full size of an individual. Furthermore, our method for sampling depth assessment was individual-based applying to the data from which the sampling was performed once. There was another model, which was sample-based and suitable for the situation which large tissues were sampled multiple times, such as in the human BCR research [29, 30]. Computational models for estimating the total richness of the repertoire could not completely overcome the limitations of the small sample, and the results were affected by the caveats of convergent recombination and experimental noise [31]. Our analysis for rarefaction and extrapolation (Additional file 4: Fig. S3 and Fig. 2) mainly assessed how closely the sequencing depth could reach the plateau of richness and reflected that our similarity measurements were performed at such an estimated sampling depth. The results also showed the differences between different isotypes and between different sample types, as well as individual differences under this method.

High sequence similarity to CDR3 is usually the standard for clonal lineage assignment of human BCR but differs among studies depending on the measurement methods. For example, to determine whether two clonotypes were somatic mutation counterparts, if both had identical V/J genes, then, the similarity to CDR3 was no more than one different amino acid [32], or within a Hamming distance (identity in fact) of nt equivalent to 95% (all sequences in a cluster had at least 80% identity) [33], or at 4–5 edits of Levenshtein distance computed from CDR3 junction nt sequences [34], or < 0.35 threshold of adjusted edit distance computed using an antibody-specific distance metric that incorporated length-normalized CDR3 edit distance, variable and joining gene use, and shared somatic mutations [35]. All of the above studies established cut-offs through systematic similarity background investigations. This kind of investigation performed in our study was in reference to the pipeline of a human antibody class switching research [36]. The heatmap of sequence identity to human BCR showed two groups of sequences, which was consistent with our result (Additional file 6: Fig. S5a–f), and supported the cut-off of 95% identity. Our data showed that this criterion was also suitable for clonal lineage establishment in the mouse BCR.

It’s worth mentioning that, although individuals of inbred mice are with narrow genetic backgrounds meaning that their self-antigen profiles are relatively consistent, as BCR repertoire is sensitive to the external antigens, the source of the animal and housing conditions have influence on the immune system. The results of this study showed the repertoire’s characteristics of a group of mice purchased from the same agency and under strictly the same breeding conditions. Besides, our results were observed from the RNA sequences of BCR IGH from total B cells reflecting the repertoire of antibodies produced. For providing a more detail view of BCR similarity among mouse individuals, the profiles of B cells subsets at different developmental stages (e.g. immature, transitional and B1 B cells) need to be further investigated.

## Conclusions

Here, we presented the landscape of the baseline BCR heavy chain repertoire in the blood and spleen of BAL and C57 mice. Our results showed that V/D/J gene usage does not significantly differ among mice, whereas the CDR3 clonotype profile is unique. Our findings emphasized the discriminate similarity levels among the IgA, IgG, and IgM isotypes and between blood and spleen samples. We showed that IgM still had tolerable CDR3 similarity, whereas IgA and IgG had worse similarity. In general, the MHSIs of CDR3 clonotypes and cross-individual clonal lineages in IgM were higher than in IgA and IgG among individual, and in the sequencing coverage of estimated clonotype richness, repertoire evenness, ratios (%) of clonotypes belonging to the somatic mutation lineage and the degree of mutation network distribution, the divergences of IgM among individuals were narrower than those of IgA and IgG. The shared CDR3 clonotypes and cross-individual clonal lineage converged more in the spleen than in the blood. We underscored the batch differences in CDR3 evenness, mutated clonotype proportions and maximal network degrees between the first and second rounds of BAL experiments. The batches of experimental animals separated in time should be carefully considered in an experimental design. In addition, a cut-off of 95% identity in the CDR3 nucleotide sequences was suitable for clonal lineage establishment. Our results improve the understanding of the nature of the antibody repertoire in BAL and C57 mice and provide a reference for designing experiments that deploy immune repertoires in mice for immunological studies.

## Methods

### Animals and sample collection

Specific pathogen-free (SPF) female BAL and C57 mice at the age 4–6 weeks were purchased from Guangdong Experimental Animal Center, Guangdong, China. After a period of acclimatization under SPF conditions for 1 week, the mice were euthanized, and peripheral blood was collected to isolate peripheral blood mononuclear cells (PBMCs) using Ficoll (Solarbio, Beijing, China). The spleens were collected to isolate splenocytes using RBC lysis buffer (Sigma-Aldrich, Shanghai, China). The animal procedures were evaluated and approved by the Laboratory Animal Ethics Committee of Guangzhou Medical University (Approval No. 2018-155). Animal experiments proceeded in strict accordance with the guidelines of the Guangdong Regulation for Administration of Laboratory Animals (2010). For technical replicates, PBMCs were isolated from all peripheral blood of an individual, placed in one test tube and divided into two parts to extract RNA separately; the entire spleen of an individual was collected to isolate splenocytes which were mixed and placed in three test tubes, two of which were used to extract RNA separately. The mouse sample size was in reference to other studies and no statistical method was used to predetermine the sample size. The investigators were not blinded to allocation during experiments and outcome assessment. This study was carried out in compliance with the ARRIVE guidelines (https://arriveguidelines.org).

### RNA extraction and cDNA synthesis

Total RNA was isolated from PBMCs and splenocytes (~ 1 × 10^6^ cells per sample) using TRIzol reagent (Life Technologies, Carlsbad, CA, USA) according to the manufacturer’s instructions. The RNA concentration was determined using a NanoDrop 2000 spectrophotometer. Total RNA (~ 50 ng) was reverse transcribed by 5' rapid amplification of cDNA ends using SMARTer PCR cDNA synthesis kits (Clontech Laboratories Inc., Mountain View, CA, USA) as described previously [37]. The reaction (10 µL) proceeded as follows. An RNA template was mixed with mouse BCR heavy chain primers (IgG, CCCTTGACMAGGCATCCYAG; IgM, CTGGTAGTTCCAGGTGAAGG; IgA, CCAGGTCACATTCATCGTGC) and dNTP. The RNA was denatured, and priming oligonucleotides were annealed at 72 °C for 3 min; the mixture was immediately placed on ice for 2 min. SMARTer IIA oligonucleotide and SMARTScribe reverse transcriptase were added for 5'-template switching and cDNA synthesis at 42 °C for 60 min, and the reaction was terminated by heating at 70 °C for 15 min.

### BCR library preparation and repertoire sequencing

A BCR library was created using two rounds of PCR amplification. The first round comprised 2 µL of cDNA, the universal primer Smart20, and the BCR IgH chain-specific primers (IgG, CCAGGGGCCAGTGGATAGAC; IgM, CCACCAGATTCTTATCAGAC; IgA, ATCAGGCAGCCGATTATCAC). The reaction was conducted as follow: 22 cycles of 95 °C for 20 s, 62 °C for 20 s, and 72 °C for 40 s. The first-round PCR products were purified using magnetic beads (Beckman Coulter Inc., Brea, CA, USA); 20% of them were included in the second round of PCR using IgH chain-specific primers (IgG, TAGACAGATGGGGSTGTYGTT; IgM, AAGACATTTGGGAAGGACTG; IgA, GTCAGTGGGTAGATGGTGGG). The reaction was performed with 22 cycles of 95 °C for 20 s, 65 °C for 20 s, and 72 °C for 20 s. The PCR products were resolved on 2% agarose gels, and bands centered at 500–700 bp were excised and purified using Qiaquick Gel Extraction kits (Qiagen, Hilden, Germany). Illumina adaptors (Illumina Inc., San Diego, CA, USA) were ligated using the NEBnext Ultra DNA Library Prep kits (New England BioLabs Inc., Ipswich, MA, USA) as described by the manufacturer. Libraries were amplified using Illumina sequencing primers with different sample barcodes. Purified PCR products were assessed via HTS using the Illumina Nova-PE250 platform. Sequencing reads of the complete dataset are available from the European Nucleotide Archive under project accession number E-MTAB-10286.

### Additional public dataset collection

Additional public datasets of the IgM IgH repertoire from the mouse spleen (BAL and C57) and blood (C57) samples were downloaded from ArrayExpress: E-MTAB-5349 [15, 25] and E-MTAB-8585 [17, 18]. We compared only samples from normal or unimmunized mice in the two datasets.

### Sequence processing and clone identification

Low-quality sequencing reads were removed; the remaining reads were classified into IgA, IgG, and IgM subsets according to the isotype-specific primers. The classification cut-off for the in-house sequencing dataset was reads with < 5 nt mismatch between the sequences of second-round PCR isotype-specific primers and 20 nt from the beginning of read 1. The cut-off for the public datasets was reads with < 6 nt mismatch between the sequence of IgM-specific primer (CGAGGGGGAAGACATTTGGG) and 20 nt in the corresponding region of read 2. For each read 2 in the public datasets, the designed overhang sequence before the primer location was trimmed. We used MiXCR (v3.0.12) [38] for PCR error correction; V, D, and J gene identification; and CDR3 region assembly. Only sequences with qualified CDR3 regions ≥ 4 aa that did not contain out-of-frame or stop codons were further analyzed. A qualified sequence was termed a clone in this study.

### Statistical analysis

The MHSI for measuring similarity between two CDR3 aa clonotype profiles was calculated as:

$$p_{i}^{l} = \frac{{n_{i}^{l} }}{{\mathop \sum \nolimits_{i} n_{i}^{l} }} p_{i}^{c} = \frac{{n_{i}^{c} }}{{\mathop \sum \nolimits_{i} n_{i}^{c} }}$$

$$MHSI = \frac{{2\mathop \sum \nolimits_{i} p_{i}^{l} p_{i}^{c} }}{{\mathop \sum \nolimits_{i} \left( {p_{i}^{l} } \right)^{2} + \mathop \sum \nolimits_{i} \left( {p_{i}^{c} } \right)^{2} }},$$

where *n*^*l*^ or *n*^*c*^ is the abundance (or number) of one unique CDR3 aa clonotype in sample *l* or sample *c*, respectively (Samples *l* and *c* are taken together to have *i* clonotypes.). Thus, *p*^*l*^ or *p*^*c*^ is the frequency of one unique CDR3 aa clonotype in its repertoire of samples *l* and *c*, respectively.

The evenness of one CDR3 aa clonotype repertoire was evaluated using the NSDE calculated as:

$$NSDE = - \mathop \sum \limits_{i = 1}^{N} \frac{{p_{i} \ln p_{i} }}{\ln N},$$

where *p*^*i*^ is the frequency of the *i*th clonotype, and *N* is the total number of clonotypes.

Rarefaction and extrapolation curves were constructed based on Chao’s estimates [30] using the iNEXT package [39] in R with q = 0 (order of Hill number), nboot = 100 (100 bootstrap replications), and endpoint = 5 (fivefold the sample size applied in extrapolation). Clonal lineage networks were constructed and node degrees were calculated using the igraph package [40] in R.

Unpaired and paired data were analyzed using Wilcoxon rank sum and signed rank tests, respectively. Values with two-sided *p* < 0.05 were considered as statistically significant. For statistical reasonableness, such as in computing the means or sums for individuals, only one result with a larger corresponding value in the technical duplication was used.

## Supplementary Information

**Additional file 1: Fig. S1.** Proportion of IgA/G/M in libraries. Pairs of technical duplications are marked with letters a and b.

**Additional file 2: Table S1.** Summary of qualified sequencing throughputs and numbers of unique clonotypes.

**Additional file 3: Fig. S2.** Heatmap of V/D/J gene usage in IgM. Gene names (**a**, V gene; **b**, D gene; **c**, J gene) are presented in vertical column on the right (D empty in **b** indicates frequency of clones in which D gene did not emerge in CDR3 region). Mouse IDs are shown at bottom of the row. Pairs of technical duplications in mice are marked letters a and b. Top three colored rows indicate samples from different groups of strains, sample types, and batches.

**Additional file 4: Fig. S3.** Curves of rarefaction analysis for CDR3 aa clonotype. Results (solid lines) were interpolated by subsampling data without replacement in 1% of total clone increments (each with 100 replications) and determining numbers of clonotypes represented by these clones. Clonotype richness (extrapolation, dashed lines) was estimated in up to ≥ fivefold observed clones based on Chao estimator formula. Data are shown as means ± SEM (light color). Results were calculated from in-house sequencing repertoires of BAL (**a**) and C57 (**b**) and E5349 and E8585 public datasets of BAL (**c**) and C57 (**d**). Isotypes, strains, and sample types are shown at top of figure. We used only one dataset with numerous clones for technical duplication.

**Additional file 5: Fig. S4.** Heatmap of MHSI. We computed MHSIs through CDR3 aa abundance of each pair of repertories in IgA (**a**), IgG (**b**), and IgM (**c**). Pairs of technical duplications in mice are marked with letters a and b. Top three colored rows indicate samples from different groups of strains, sample types, and batches.

**Additional file 6: Fig. S5.** Similarity distribution of clonotype nearest neighbor. Similarity is ratio (%) of identical nt between CDR3 nt clonotypes and their nearest neighbors. Results were computed for IgA (**a** and **b**), IgG (**c** and **d**) and IgM (**e** and **f**) using our in-house sequencing data and IgM of E5349 and E8585 datasets (**g** and **h**). We used one dataset with numerous CDR3 nt clonotypes for technical duplication.

**Additional file 7: Fig. S6.** The distribution of node numbers in cross-individual clonal lineages. The result was computed from our in-house sequencing data. One dataset with numerous CDR3 nt clonotypes was used for technical duplication.

**Additional file 8: Table S2.** Information of cross-individual clonal lineages and their nodes.

**Additional file 9: Fig. S7.** Repertoire similarity measured by cross-individual clonal lineages with no less than 5 nodes. The percentage distributions of lineages appeared in different number of blood and spleen samples were calculated from in-house sequencing data of BAL (**a**) and C57 (**b**). For the clarity of the color display, the percentages of small sample numbers are omitted (grey squares in **a** and **b**). Different colors of the dots indicate the MHSI values were computed from the same mouse or different mice (**c**). One dataset with numerous CDR3 nt clonotypes was used for technical duplication.

## Data Availability

The datasets generated for this study can be found in ArrayExpress under the project accession number E-MTAB-10286.

## Abbreviations

BCR: B cell receptor
IgH: Immunoglobulin heavy chain
CDR: Complementarity determining region
aa: Amino acid
nt: Nucleotide
BAL: BALB/c mouse strain
C57: C57BL/6J mouse strain
SPF: Specific pathogen-free
PBMC: Peripheral blood mononuclear cell
HTS: High-throughput sequencing
MHSI: Morisita-Horn similarity index
NSDE: Normalized Shannon diversity entropy
