# Supplementary figures and images for: Similarity measurements of B cell receptor repertoire in baseline mice showed spectrum convergence of IgM
Source: BMC Immunol. 2022 Mar 4;23:11. doi: 10.1186/s12865-022-00482-8 (PMC8895918; doi:10.1186/s12865-022-00482-8)

IgA IgG IgM Unmatch

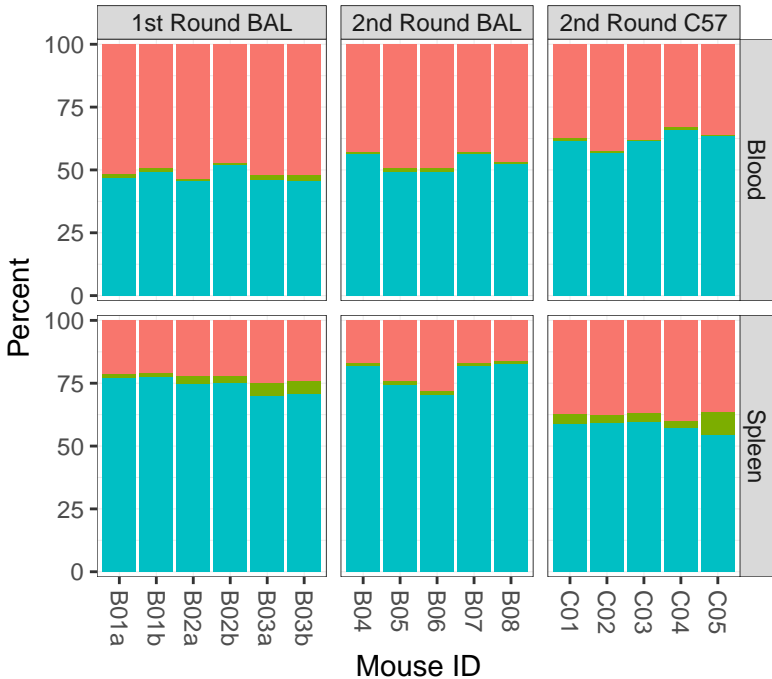

Supplement: Supplementary file 1 — Additional file 1: Fig. S1. Proportion of IgA/G/M in libraries. Pairs of technical duplications are marked with letters a and b. [file 12865_2022_482_MOESM1_ESM.pdf]

**A**

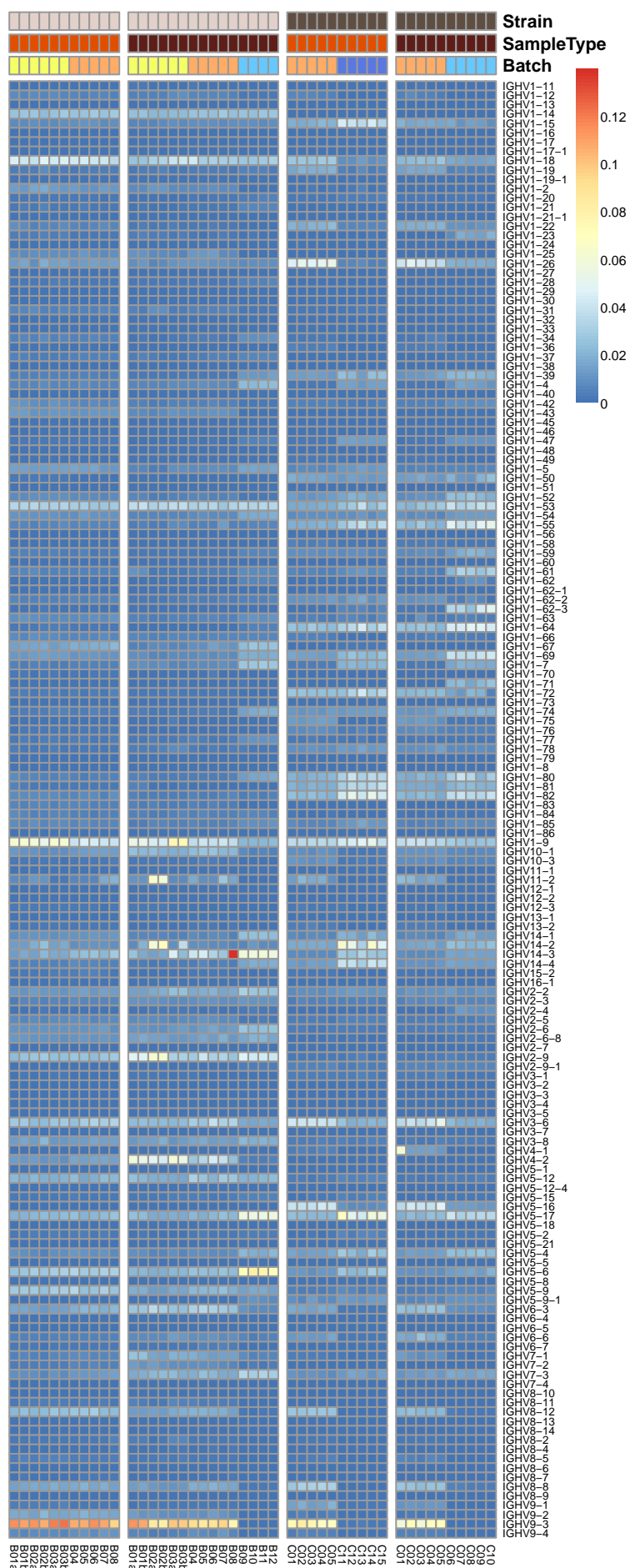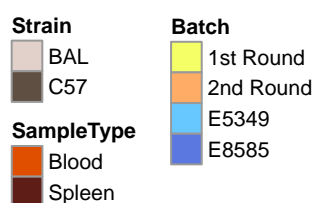

# B

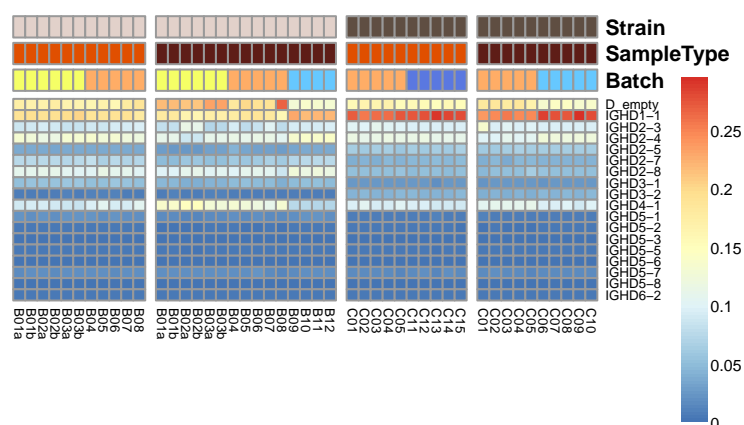

# C

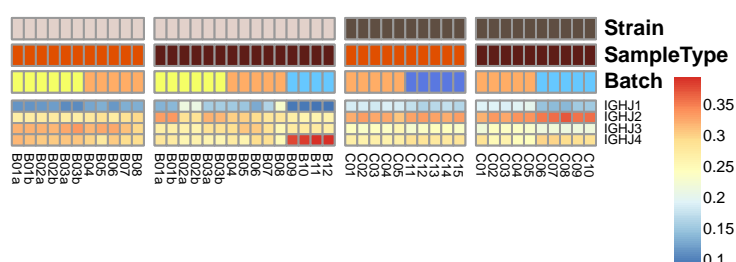

Supplement: Supplementary file 3 — Additional file 3: Fig. S2. Heatmap of V/D/J gene usage in IgM. Gene names (a, V gene; b, D gene; c, J gene) are presented in vertical column on the right (D empty in b indicates frequency of clones in which D gene did not emerge in CDR3 region). Mouse IDs are shown at bottom of the row. Pairs of technical duplications in mice are marked letters a and b. Top three colored rows indicate samples from different groups of strains, sample types, and batches. [file 12865_2022_482_MOESM3_ESM.pdf]

**A**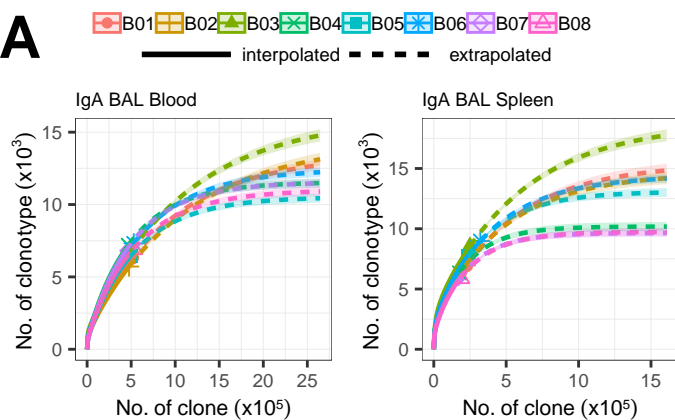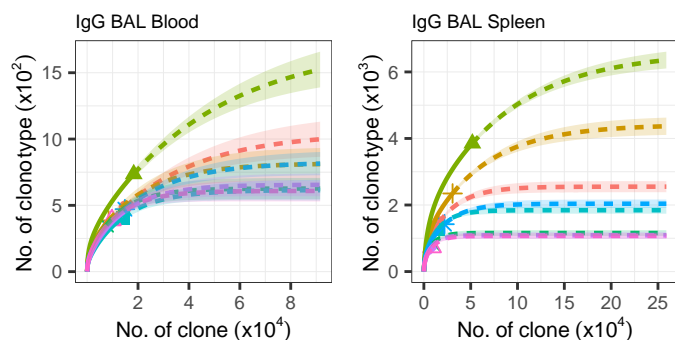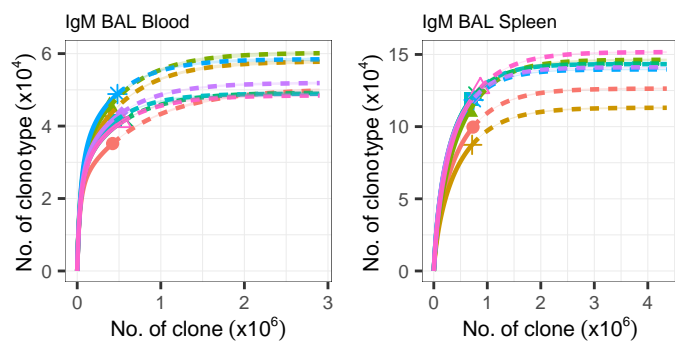**B**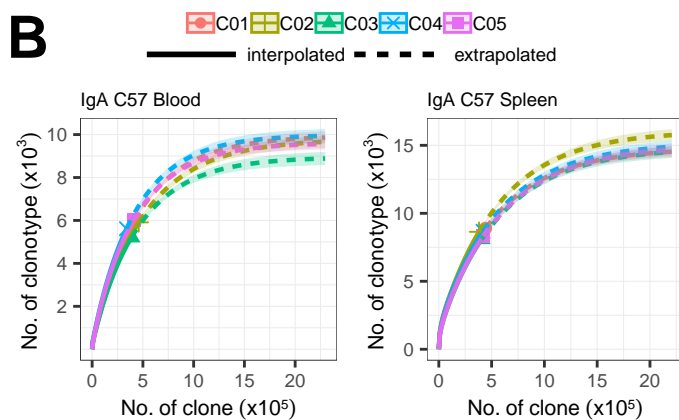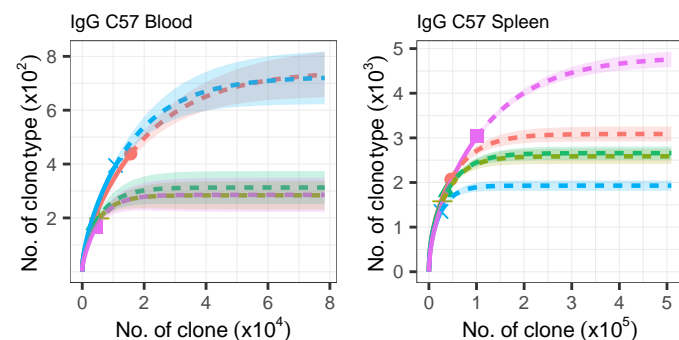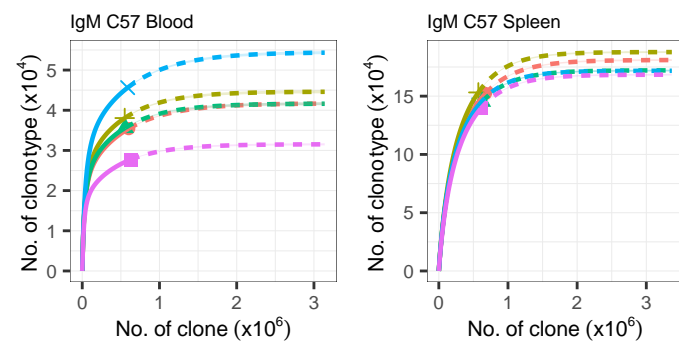**C**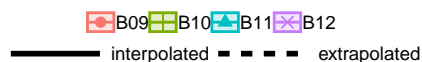**D**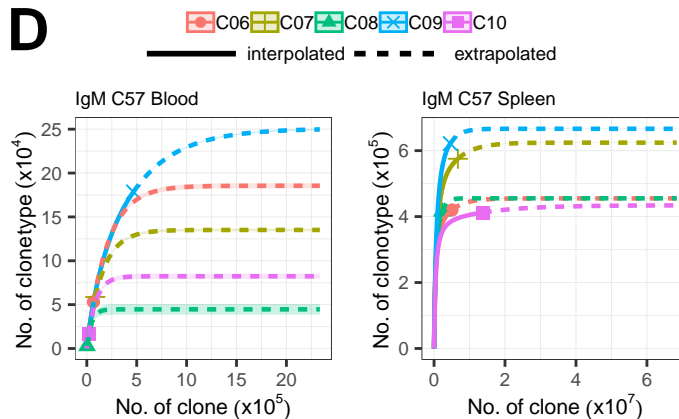

Supplement: Supplementary file 4 — Additional file 4: Fig. S3. Curves of rarefaction analysis for CDR3 aa clonotype. Results (solid lines) were interpolated by subsampling data without replacement in 1% of total clone increments (each with 100 replications) and determining numbers of clonotypes represented by these clones. Clonotype richness (extrapolation, dashed lines) was estimated in up to ≥ fivefold observed clones based on Chao estimator formula. Data are shown as means ± SEM (light color). Results were calculated from in-house sequencing repertoires of BAL (a) and C57 (b) and E5349 and E8585 public datasets of BAL (c) and C57 (d). Isotypes, strains, and sample types are shown at top of figure. We used only one dataset with numerous clones for technical duplication. [file 12865_2022_482_MOESM4_ESM.pdf]

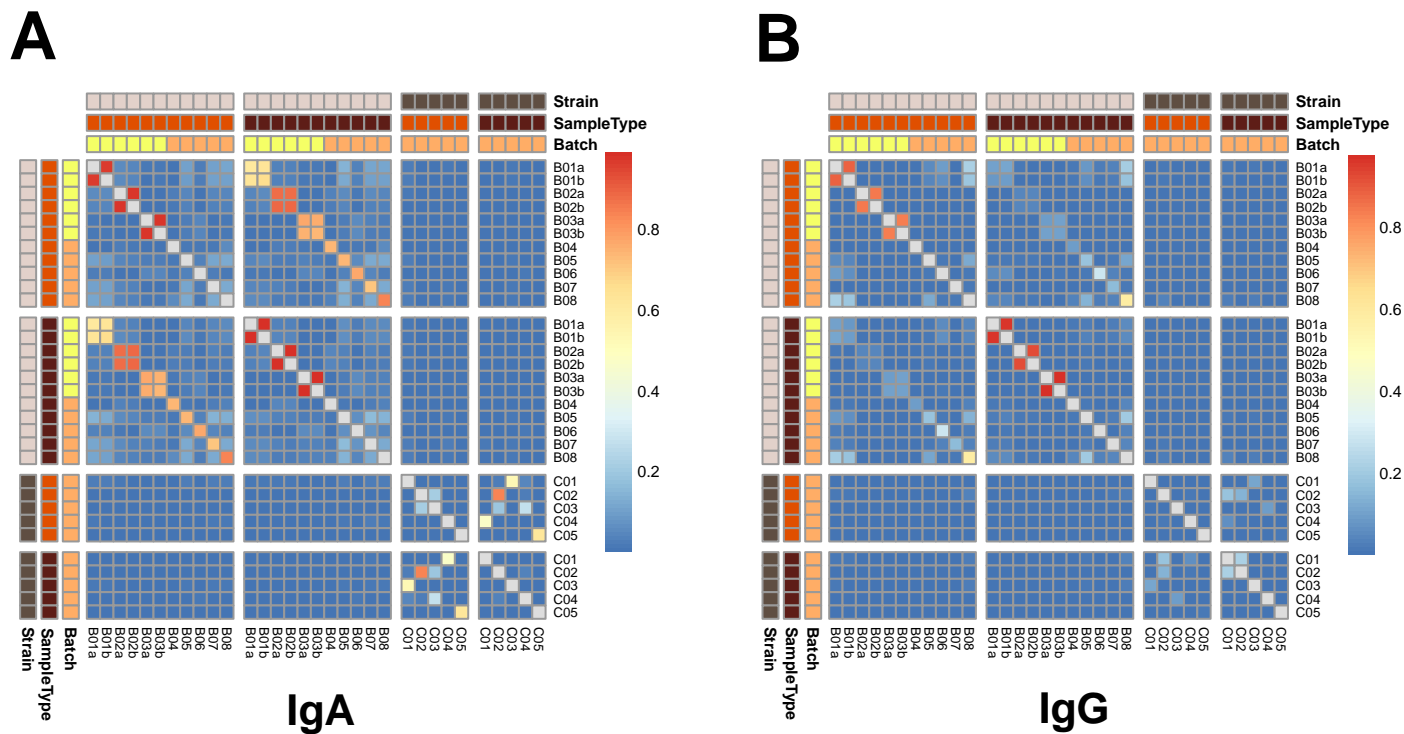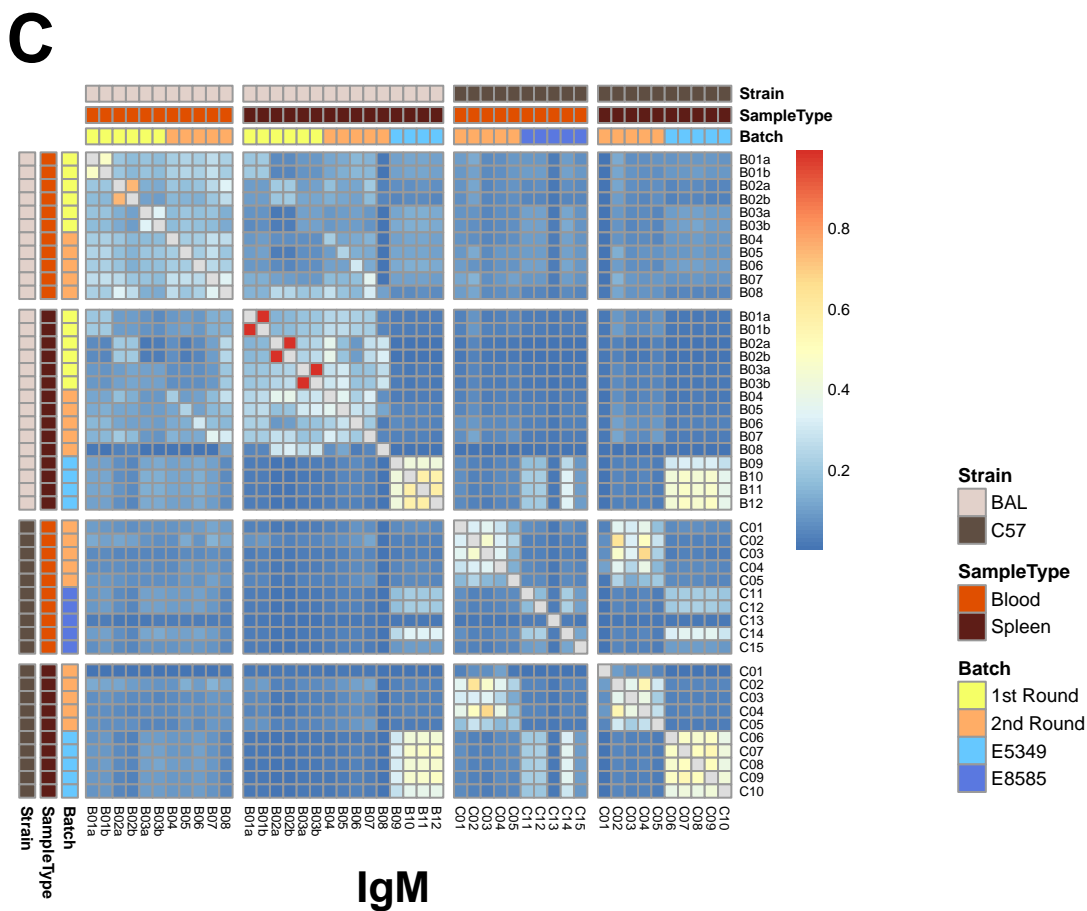

Supplement: Supplementary file 5 — Additional file 5: Fig. S4. Heatmap of MHSI. We computed MHSIs through CDR3 aa abundance of each pair of repertories in IgA (a), IgG (b), and IgM (c). Pairs of technical duplications in mice are marked with letters a and b. Top three colored rows indicate samples from different groups of strains, sample types, and batches. [file 12865_2022_482_MOESM5_ESM.pdf]

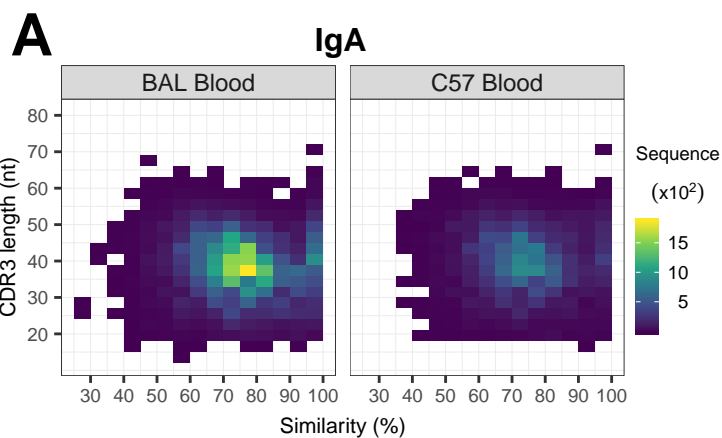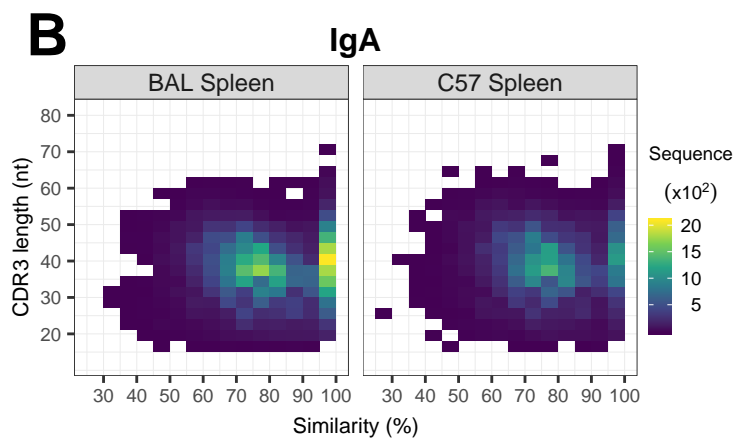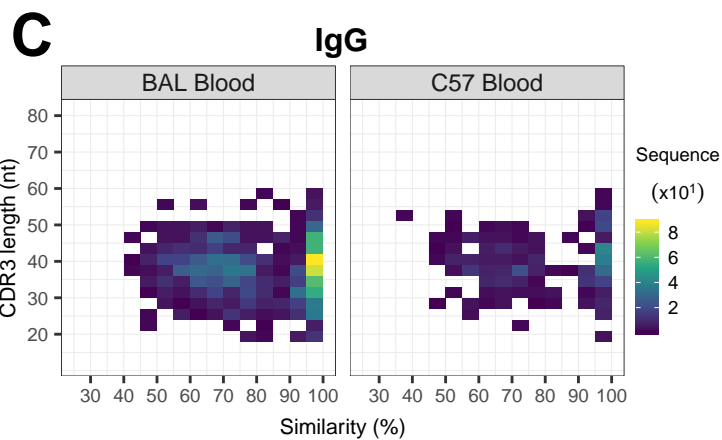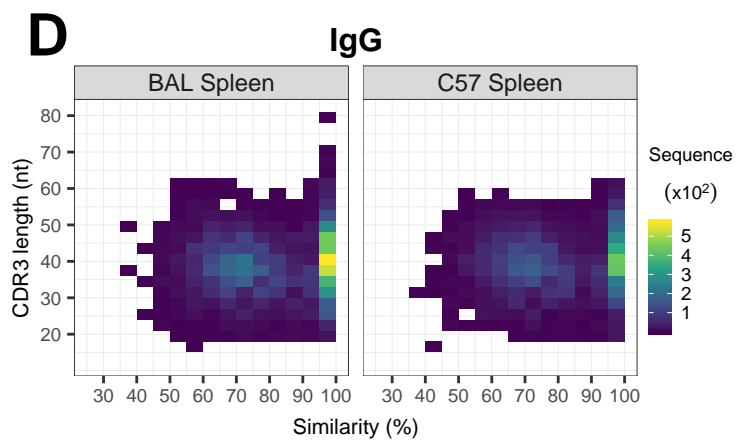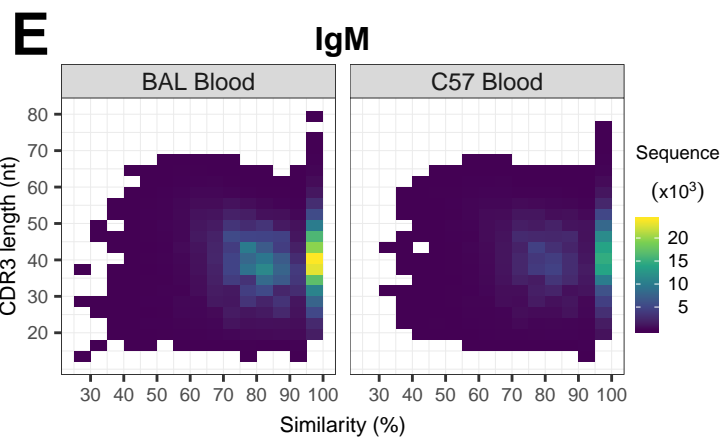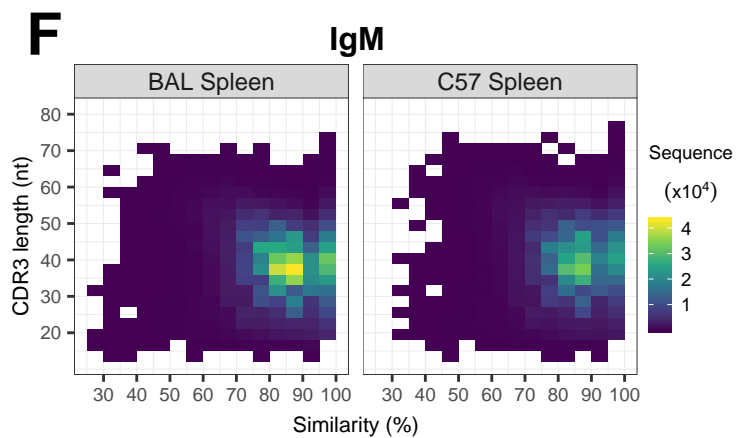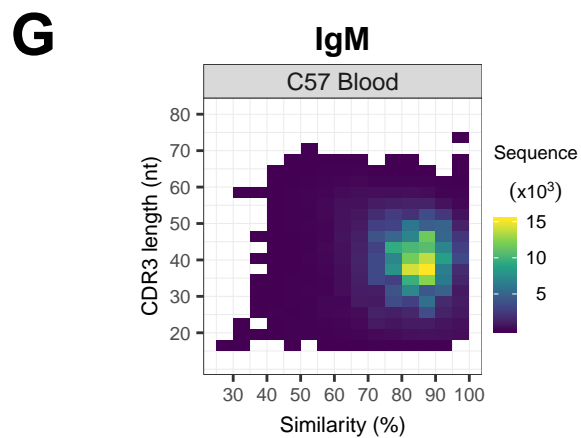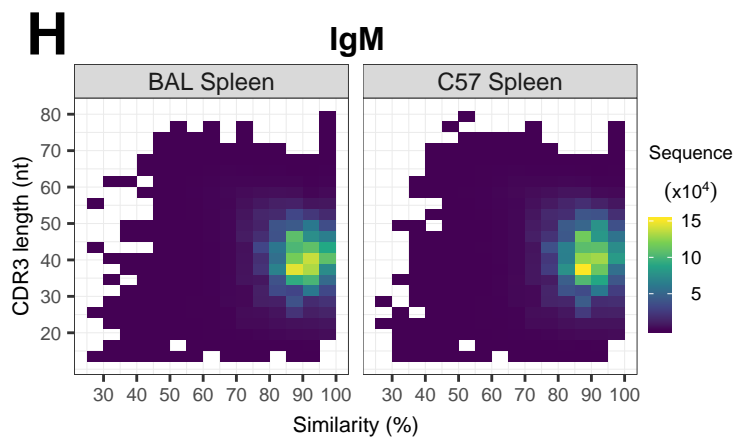

Supplement: Supplementary file 6 — Additional file 6: Fig. S5. Similarity distribution of clonotype nearest neighbor. Similarity is ratio (%) of identical nt between CDR3 nt clonotypes and their nearest neighbors. Results were computed for IgA (a and b), IgG (c and d) and IgM (e and f) using our in-house sequencing data and IgM of E5349 and E8585 datasets (g and h). We used one dataset with numerous CDR3 nt clonotypes for technical duplication. [file 12865_2022_482_MOESM6_ESM.pdf]

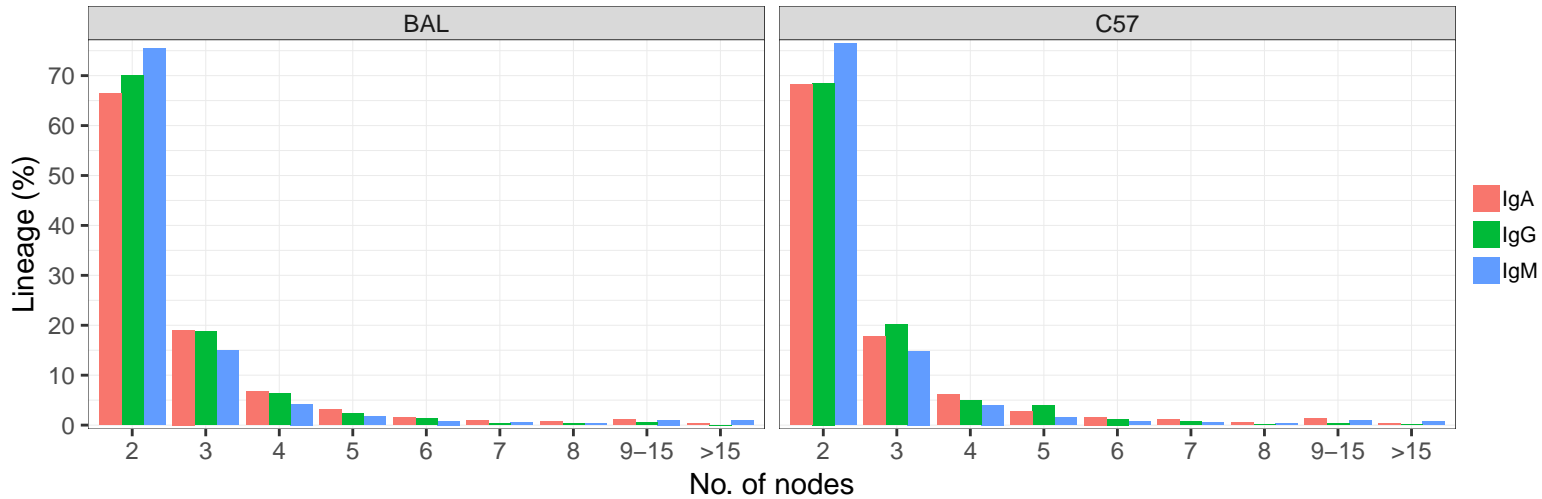

Supplement: Supplementary file 7 — Additional file 7: Fig. S6. The distribution of node numbers in cross-individual clonal lineages. The result was computed from our in-house sequencing data. One dataset with numerous CDR3 nt clonotypes was used for technical duplication. [file 12865_2022_482_MOESM7_ESM.pdf]

**A**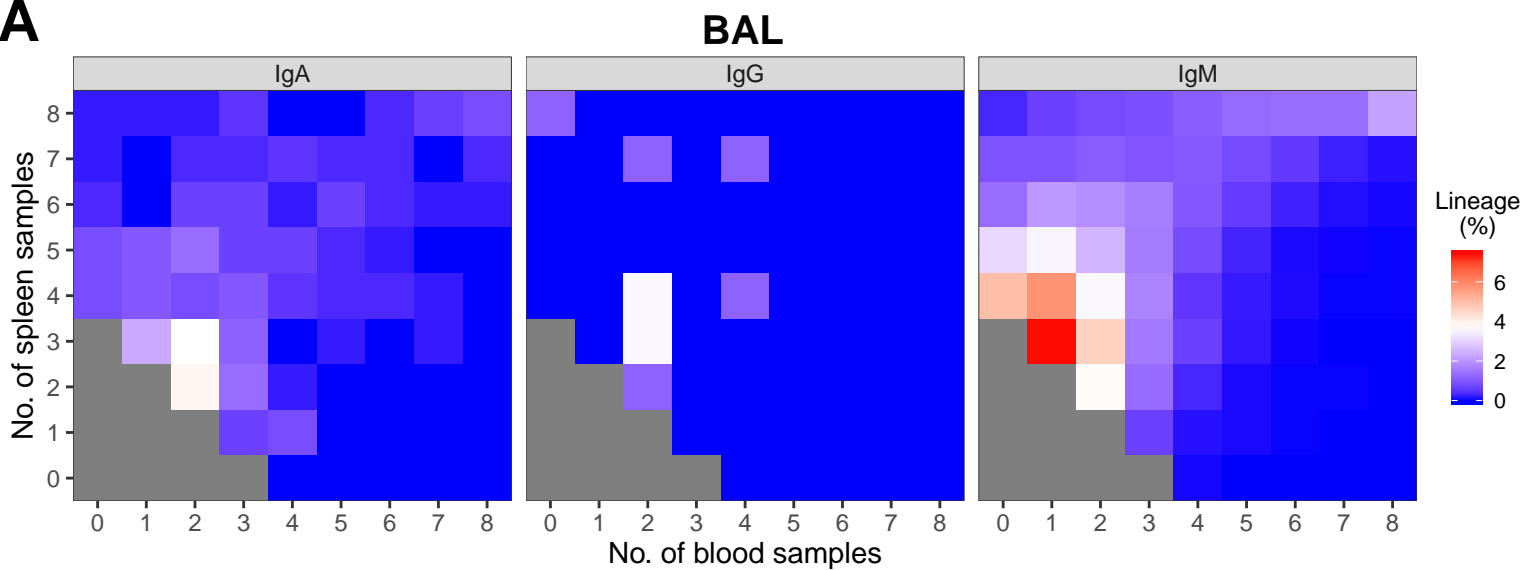**B**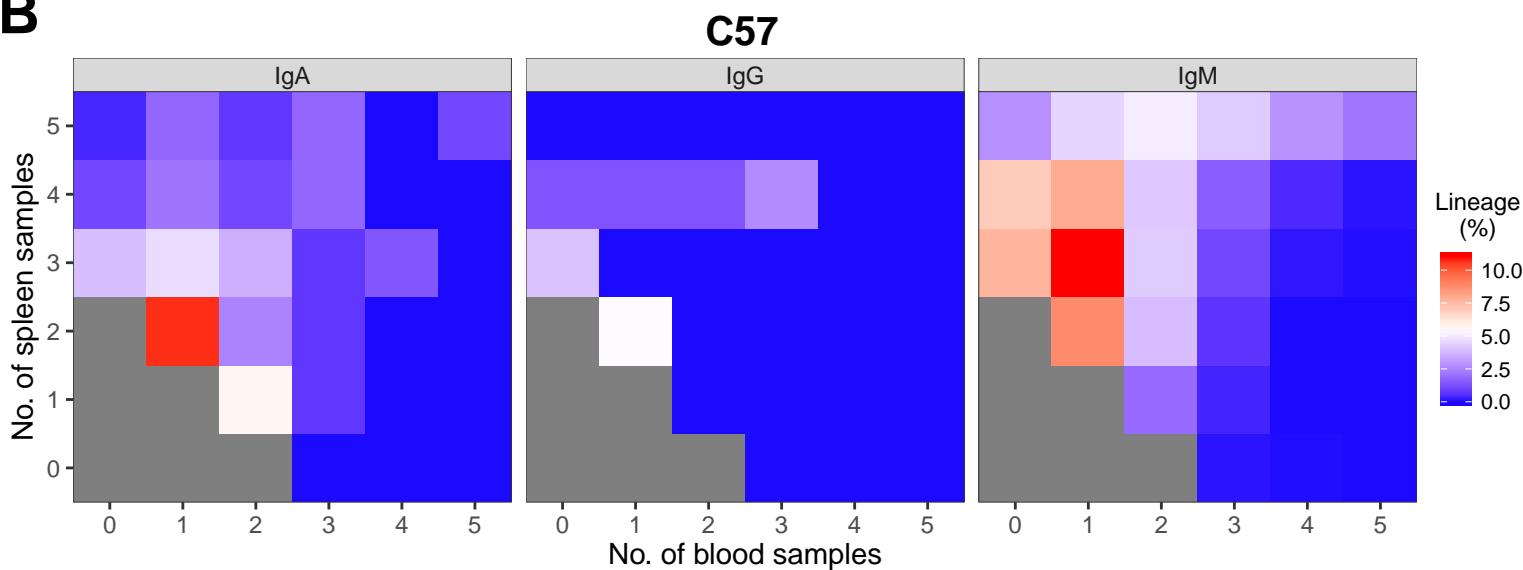**C**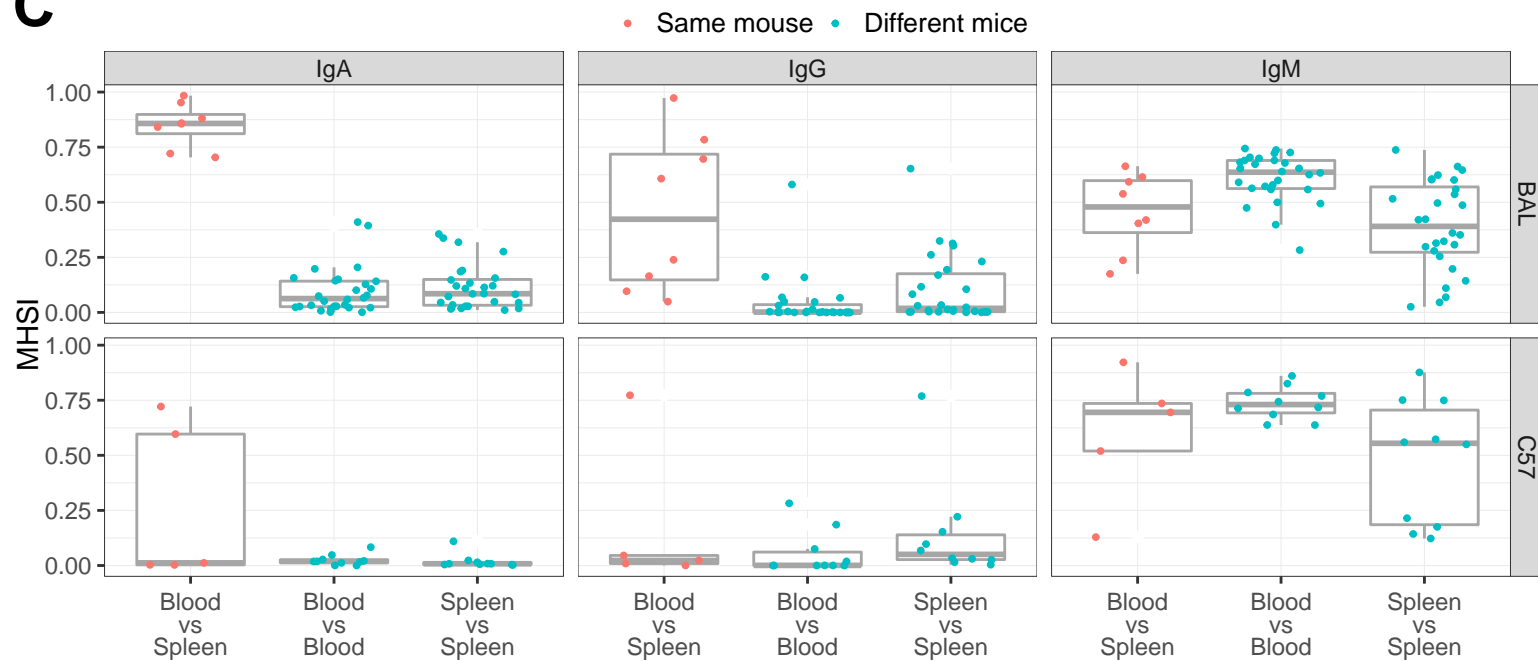

Supplement: Supplementary file 9 — Additional file 9: Fig. S7. Repertoire similarity measured by cross-individual clonal lineages with no less than 5 nodes. The percentage distributions of lineages appeared in different number of blood and spleen samples were calculated from in-house sequencing data of BAL (a) and C57 (b). For the clarity of the color display, the percentages of small sample numbers are omitted (grey squares in a and b). Different colors of the dots indicate the MHSI values were computed from the same mouse or different mice (c). One dataset with numerous CDR3 nt clonotypes was used for technical duplication. [file 12865_2022_482_MOESM9_ESM.pdf]
